# Indeterminacy of cannabis impairment and ∆^9^-tetrahydrocannabinol (∆^9^-THC) levels in blood and breath

**DOI:** 10.1038/s41598-022-11481-5

**Published:** 2022-05-18

**Authors:** Gregory T. Wurz, Michael W. DeGregorio

**Affiliations:** 1 RCU Labs, Inc., 408 Sunrise Avenue, Roseville, CA 95661-4123 USA; 2 Cancer Immunotherapy Research Institute, 408 Sunrise Avenue, Roseville, CA 95661 USA

**Keywords:** Pharmacodynamics, Translational research

## Abstract

Previous investigators have found no clear relationship between specific blood concentrations of ∆^9^-tetrahydrocannabinol (∆^9^-THC) and impairment, and thus no scientific justification for use of legal “per se” ∆^9^-THC blood concentration limits. Analyzing blood from 30 subjects showed ∆^9^-THC concentrations that exceeded 5 ng/mL in 16 of the 30 subjects following a 12-h period of abstinence in the absence of any impairment. In blood and exhaled breath samples collected from a group of 34 subjects at baseline prior to smoking, increasing breath ∆^9^-THC levels were correlated with increasing blood levels (*P* < 0.0001) in the absence of impairment, suggesting that single measurements of ∆^9^-THC in breath, as in blood, are not related to impairment. When post-smoking duration of impairment was compared to baseline ∆^9^-THC blood concentrations, subjects with the highest baseline ∆^9^-THC levels tended to have the shortest duration of impairment. It was further shown that subjects with the shortest duration of impairment also had the lowest incidence of horizontal gaze nystagmus at 3 h post-smoking compared to subjects with the longest duration of impairment (*P* < 0.05). Finally, analysis of breath samples from a group of 44 subjects revealed the presence of transient cannabinoids such as cannabigerol, cannabichromene, and ∆^9^-tetrahydrocannabivarin during the peak impairment window, suggesting that these compounds may be key indicators of recent cannabis use through inhalation. In conclusion, these results provide further evidence that single measurements of ∆^9^-THC in blood, and now in exhaled breath, do not correlate with impairment following inhalation, and that other cannabinoids may be key indicators of recent cannabis inhalation.

## Introduction

Finding an objective measure of recent cannabis use that correlates with impairment has proven to be an elusive goal. In the United States, where the recreational use of cannabis has been legalized in 18 states and Washington, D.C. as of early 2022, some of these states have resorted to setting per se legal limits for ∆^9^-tetrahydrocannabinol (∆^9^-THC) concentrations in blood, limits above which test subjects are considered to be legally impaired. For example, Illinois, Montana, and Washington have established a per se limit of 5 ng/mL, while Nevada and Ohio use a limit of 2 ng/mL^1^. In Colorado, a 5-ng/mL permissible inference standard is employed^1^, meaning that a jury can legally presume that subjects testing at or above this level were impaired, unless evidence to the contrary can be provided by the defense. Published research in the last several years, however, has shown that there is no clear relationship between specific blood or oral fluid concentrations of ∆^9^-THC and impairment^2–6^. In other words, there is currently no scientific justification for the use of per se legal limits for ∆^9^-THC blood concentrations, leaving cannabis users in these states at risk of being wrongfully prosecuted for driving under the influence (DUI) of cannabis.

Exhaled breath has emerged as a potential alternative test matrix to blood and oral fluid for establishing recent cannabis use within the impairment window. While it has been known for nearly 40 years that ∆^9^-THC can be detected in breath^7^, only relatively recently has this matrix been explored for assessing recent cannabis use and impairment. Exhaled breath testing for recent cannabis use is predicated on a short period of detection for ∆^9^-THC within the impairment window, or approximately two to three hours. A study by Himes et al. suggested that ∆^9^-THC is generally detectable in breath for only about two hours after smoking even in chronic users^8^; however, more recent studies have shown that ∆^9^-THC can remain detectable in the breath up to several days following most recent use^9,10^.

Our recent publication describes the development of a new test for recent cannabis use and impairment based on two-point breath sampling, with or without a one-point confirmatory blood test, that can accurately detect whether a subject used cannabis through inhalation within the three-hour impairment window with no false positive results^11^. During that study, blood and exhaled breath samples were collected at baseline prior to smoking and at various time points up to four hours post-smoking and then analyzed by liquid chromatography high-resolution mass spectrometry (LC-HRMS) for ∆^9^-THC and other cannabinoids. Impairment was evaluated through subject self-assessments as well as through physical assessments of horizontal gaze nystagmus (HGN). Nystagmus refers to the involuntary movement or jerking of the eyes as they gaze to either side (horizontal) or up and down (vertical), and it is a component of standardized field sobriety testing^12^. Someone experiencing nystagmus is unaware of its occurrence. Using the data from our recent study^11^, here we present evaluations of: (1) pre-smoking ∆^9^-THC blood concentrations in relation to currently used per se legal limits; (2) post-smoking duration of impairment compared to baseline ∆^9^-THC blood concentrations; (3) duration of impairment compared to the incidence of HGN; (4) the relationship between ∆^9^-THC concentrations at baseline and at peak impairment in exhaled breath and blood; and (5) key cannabinoids detected in breath during the impairment window to assess the utility of single-point analyses of ∆^9^-THC in blood and exhaled breath for detecting recent cannabis use within the impairment window.

## Results

### Clinical study: subject demographics

A total of 74 subjects were recruited over a one-year period, the majority of whom were chronic daily cannabis users. For most subjects, smoking and/or vaping was the primary route of use, while some subjects also reported use of cannabis edibles. When asked about frequency of use, most subjects reported daily use. There was an approximate 3:1 ratio of males to females, and the subjects ranged in age from 21 to 42 years, with an average age of 25.0 years. The subjects reported a mean cannabis use history of 9.0 years. See Table 1 for complete subject demographic information. Table 2 shows the ∆^9^-THC content of the different chemovars used in the study, which subjects received them, and the maximum possible ∆^9^-THC dose.

**Table 1:** Clinical subject demographics.

Age (years)	Sex	Route of administration^a^	Frequency of use (# days/last 14 days)^a^	Cannabis use history (years)^a^
Average (± SD) 25.0 ± 4.5	Male 56 (75.7%)	Inhalation 58 (78.4%)	Average (± SD) 11.9 ± 4.1	Average (± SD) 9.0 ± 4.4
Median 23	Female 18 (24.3%)	Inhaled/Edibles 8 (10.8%)	Median 14	Median 9
		Edibles 2 (2.7%)		

^a^Six subjects (8.1%) did not report route of administration, frequency of use, or use history.

**Table 2:** The **∆**^9^-THC content information on cannabis chemovars smoked by study subjects.

Chemovar	∆^9^-THC Content (% by weight)	Maximum ∆^9^-THC Dose (mg)^a^	Used by subjects #
1	19.3	96.5	1–8
2	21.4	107	9–15
3	24.4	122	16–22
4	21.3	106.5	23–29
5	21.5	107.5	30–36
6	23.9	119.5	37–40
7	17.4	87	41–46
8	25.0	125	47–51
9	28.38	141.9	52, 54–56
10	29.0	145	57–64
11	8.51	42.5	53, 65
12	23.0	115	66–70
13	24.61	123	71–74

^a^Based on a 500-mg cigarette.

### ∆^9^-THC blood concentrations are not related to self-assessed impairment

**Figure 1:**
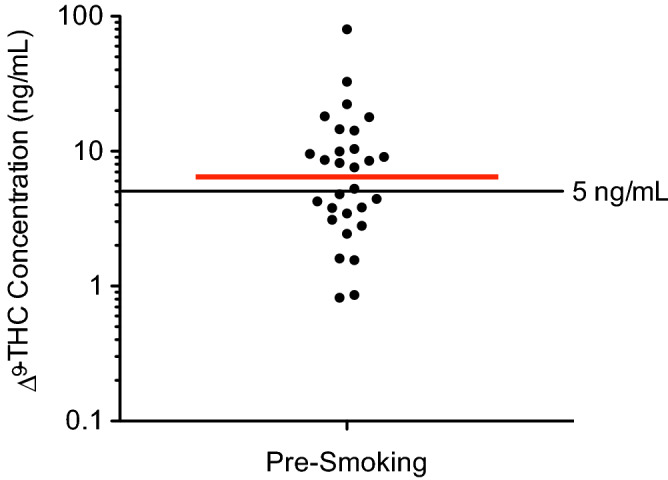
Baseline ∆^9^-THC blood concentrations in a group of 30 subjects. Pre-smoking (baseline) ∆^9^-THC blood concentrations were evaluated by LC-HRMS in a group of 30 subjects prior to smoking a 500-mg cannabis cigarette. The horizontal red bar indicates the median concentration (6.4 ng/mL), and the horizontal black bar at 5 ng/mL indicates a common legal per se ∆^9^-THC blood concentration limit. One subject not shown (∆^9^-THC not detected).

As shown in Fig. 1, ∆^9^-THC blood concentrations measured prior to smoking in subjects #1–30 exceeded 5 ng/mL (currently the legal limit in Illinois, Montana, and Washington) in 16 subjects (53.3%) in our study, and 25 subjects (83.3%) had ∆^9^-THC concentrations that exceeded 2 ng/mL (the legal limit in Nevada and Ohio), in the absence of impairment as determined by subject self-assessments. We previously showed that self-assessed impairment data corresponded well with evaluations of HGN used as a means of physically assessing impairment^11^.

### Average percent of maximum impairment over time

**Figure 2:**
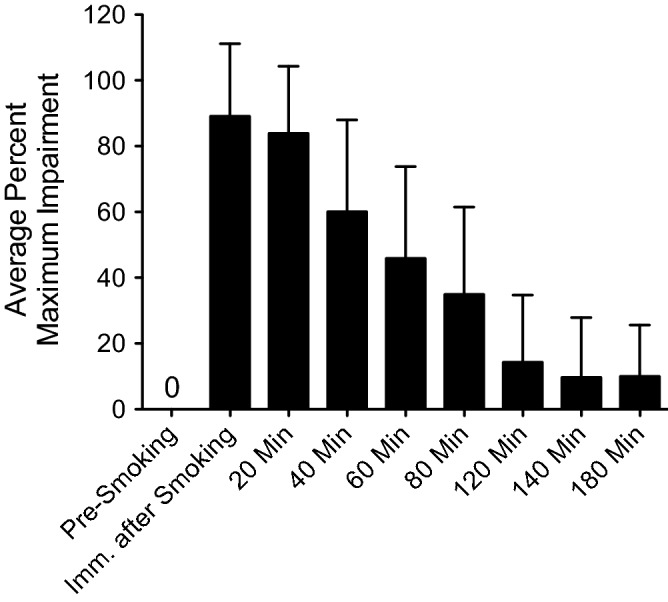
Average percent maximum self-assessed impairment (+ SD) from pre-smoking to three hours post-smoking (*N* = 74). For each subject, impairment data were expressed as a percentage relative to the individual subject’s maximum self-reported impairment level.

Figure 2 shows the average percent of maximum self-assessed impairment over time up to three hours post-smoking for all 74 subjects. The average percent of maximum impairment peaked within the first 20 min after smoking, and by three hours post-smoking it had fallen to approximately 10%, which is consistent with an approximate three-hour window of impairment.

### Duration of impairment inversely related to pre-smoking ∆^9^-THC concentrations in blood

**Figure 3:**
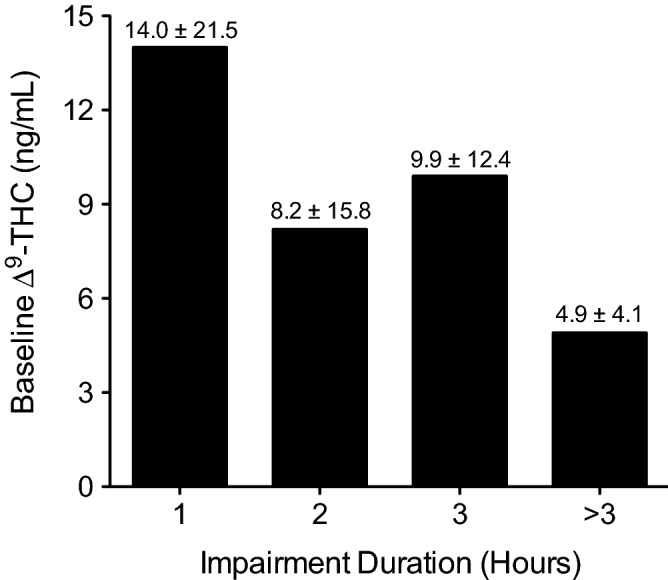
Post-smoking duration of impairment compared to baseline ∆^9^-THC blood concentration in 64 subjects. As determined by self-assessment, subjects were stratified by duration of impairment [1 h (*N* = 19), 2 h (*N* = 24), 3 h (*N* = 17), > 3 h (*N* = 4)] after smoking a 500-mg cannabis cigarette. Mean ∆^9^-THC concentration (± SD) is shown above each bar.

Pre-smoking ∆^9^-THC concentrations were evaluated in a total of 64 subjects (#1–51, #62–74), who were then stratified by their duration of impairment after smoking: 1 h; 2 h; 3 h; > 3 h. There were 10 subjects (#52–61) from whom blood samples were not collected prior to smoking. Duration of impairment was based on subject self-assessments performed prior to smoking and at various time points post-smoking using a 10-point scale. Some subjects did not finish smoking their cannabis cigarettes because they considered themselves completely impaired; however, all subjects experienced some impairment to the level where they felt they could no longer drive, which was the desired effect, but they were not maximally impaired. As shown in Fig. 3, after stratification, the duration of impairment post-smoking appeared to be inversely related to pre-smoking ∆^9^-THC blood concentrations, suggesting that subjects with the highest baseline ∆^9^-THC concentrations, indicative of chronic use, tended to have the shortest duration of impairment after smoking. Prior to stratification, a correlation analysis found no significant relationship (*P* = 0.4736) between baseline ∆^9^-THC blood concentrations and the duration of impairment, which was likely due to the high degree of variation in baseline ∆^9^-THC levels.

### Relationship between duration of impairment and the incidence of HGN

**Figure 4:**
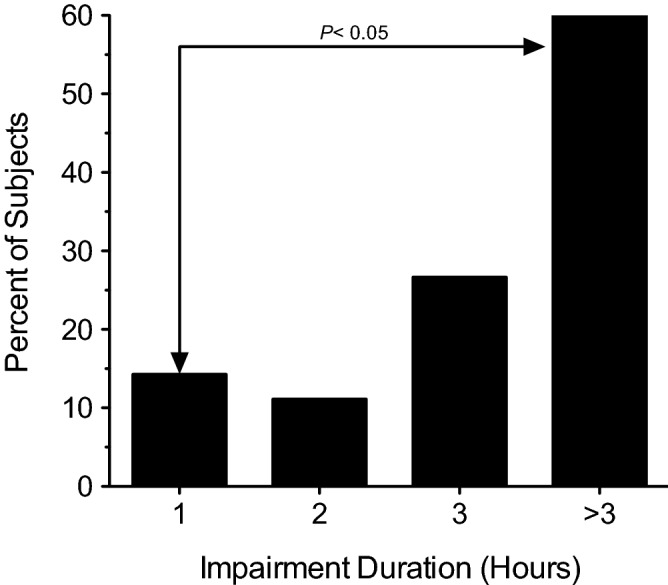
Relationship between duration of impairment and the incidence of nystagmus. A total of 44 subjects were assessed for nystagmus prior to smoking and at various time points up to three hours post-smoking (43/44 subjects were evaluated at three hours post-smoking). As determined by self-assessments, subjects were stratified by duration of impairment, 1 h (*N* = 14), 2 h (*N* = 9), 3 h (*N* = 15), or > 3 h (*N* = 5), and the incidence of nystagmus at three hours post-smoking was calculated.

A total of 44 subjects were evaluated for the presence of HGN as a means of physically assessing impairment prior to smoking and at various time points up to three hours post-smoking. After stratifying the subjects by duration of impairment as shown in Fig. 4, the duration of impairment was compared to the incidence of HGN at three hours post-smoking, at which time all but one subject (43/44) were evaluated. The results showed that subjects with the shortest duration of impairment tended to have the lowest incidence of HGN at three hours post-smoking. After performing an independent samples t-test, subjects with a 1-h duration of impairment (*N* = 14) were found to have a significantly lower incidence of HGN (*P* = 0.0491) at three hours post-smoking compared to subjects with a duration of impairment > 3 h (*N* = 5) (see Fig. 4).

### Relationship between baseline ∆^9^-THC concentrations in exhaled breath and blood

A significant correlation (*P* < 0.0001) was observed between increasing baseline ∆^9^-THC concentrations in blood and increasing concentrations in exhaled breath in 34 subjects from whom both breath and blood samples were collected prior to smoking, in the absence of impairment, as shown in Table 3. In 10 of the 44 subjects from whom both breath and blood samples were collected, no baseline samples were taken. Those subjects found to have the lowest ∆^9^-THC levels in their breath (undetectable; *N* = 11) tended to have the lowest corresponding average concentration in blood, while subjects who had the highest average ∆^9^-THC breath concentration also tended to have the highest average concentration in blood (see Table 3). For the 23 subjects who had detectable ∆^9^-THC in breath, their corresponding blood concentrations were stratified into two groups: ∆^9^-THC < 20 ng/mL (*N* = 20) and ∆^9^-THC > 20 ng/mL (*N* = 3).

**Table 3:** Relationship between baseline ∆^9^-THC concentrations in exhaled breath and blood (*N* = 34).

Subject category	Average ∆^9^-THC concentrations (± SD)	
	Breath (ng/filter)	Blood (ng/mL)
Undetectable baseline ∆^9^-THC in breath	Not detected (*N* = 11)	2.8 ± 1.5 (*N* = 11)
Baseline blood ∆^9^-THC < 20 ng/mL	0.2 ± 0.3 (*N* = 20)	3.5 ± 2.8 (*N* = 20)
Baseline blood ∆^9^-THC > 20 ng/mL	1.5 ± 2.0 (*N* = 3)	54.1 ± 29.5 (*N* = 3)

### Relationship between ∆^9^-THC concentrations in exhaled breath and blood at peak impairment

**Figure 5:**
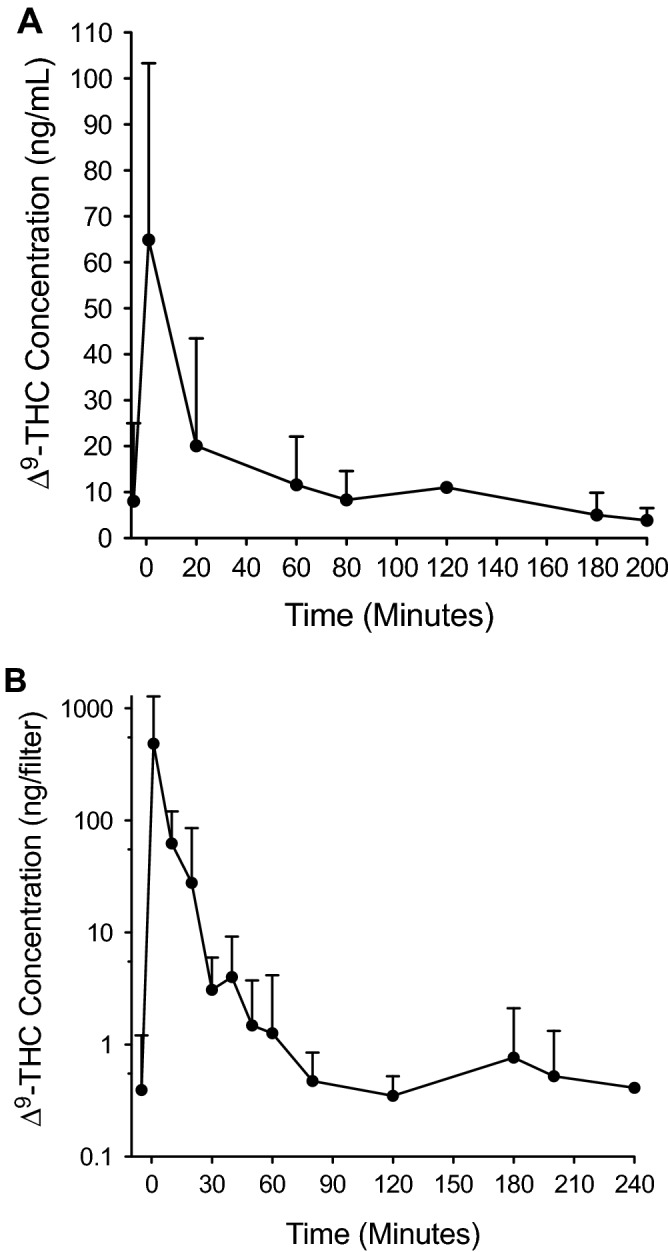
Relationship between blood and breath ∆^9^-THC concentrations before and after smoking in 34 subjects. Average (+ SD) ∆^9^-THC concentrations are shown in (A) blood (ng/mL) and (B) breath (ng/filter). Lack of error bars indicates *N* = 2. The number of subjects at each time point varies due to non-detection (ND) of ∆^9^-THC, no sample (NS) collected, and removal of outliers. For blood, *N* = 32 prior to smoking (2 ND), 24 at 1 min post-smoking (10 NS), 25 at 20 min (9 NS), 33 at 60 min (1 outlier), 8 at 80 min (24 NS, 1 ND, 1 outlier), 2 at 120 min (32 NS), 22 at 180 min (8 NS, 2 ND, 2 outliers), and 12 at 200 min (21 NS, 1 ND). For breath, *N* = 23 prior to smoking (11 ND), 24 at 1 min post-smoking (9 NS, 1 outlier), 11 at 10 min (23 NS), 32 at 20 min (2 outliers), 11 at 30 min (23 NS), 20 at 40 min (14 NS), 11 at 50 min (23 NS), 25 at 60 min (9 NS), 10 at 80 min (23 NS, 1 ND), 3 at 120 min (31 NS), 26 at 180 min (7 NS, 1 ND), 3 at 200 min (30 NS, 1 ND), and 2 at 240 min (32 NS).

The relationship between increasing ∆^9^-THC concentrations in blood and exhaled breath observed at baseline prior to smoking was also observed post-smoking at the time of peak impairment in the same group of 34 subjects. As shown in Table 4, after stratifying the samples based on detectable ∆^9^-THC in breath and blood prior to smoking (undetectable in breath, < 20 ng/mL in blood at baseline, and > 20 ng/mL in blood at baseline), subjects who had the highest ∆^9^-THC concentrations in breath and blood at baseline also tended to have the highest concentrations at peak impairment. Prior to stratification, no significant correlation (*P* = 0.2297) was found between increasing ∆^9^-THC concentrations in breath and blood at peak impairment in the 27 subjects who had matching breath and blood samples collected at peak impairment. As previously reported^11^, peak impairment occurred within the first hour after smoking in all subjects based on self-assessments. Together, the data in Tables 3 and 4 show that while individual measurements of ∆^9^-THC in exhaled breath cannot be reliably associated with impairment, higher ∆^9^-THC concentrations in breath both before and after smoking not surprisingly tend to be associated with higher blood concentrations. Figure 5 shows the relationship between breath and blood ∆^9^-THC concentrations before and after smoking, with concentrations peaking within the first 20 min post-smoking and then rapidly declining to near baseline levels 3–4 h post-smoking.

**Table 4:** Relationship between ∆^9^-THC concentrations in exhaled breath and blood at peak impairment.

Subject category	Average ∆^9^-THC concentrations (± SD)	
	Breath (ng/filter)	Blood (ng/mL)
Undetectable baseline ∆^9^-THC in breath	403 ± 984 (*N* = 10)^a^	51.9 ± 24.3 (*N* = 5)^b^
Baseline blood ∆^9^-THC < 20 ng/mL	217 ± 317 (*N* = 20)	56.3 ± 46.3 (*N* = 16)^c^
Baseline blood ∆^9^-THC > 20 ng/mL	1070 ± 439 (*N* = 3)	95.0 ± 29.7 (*N* = 3)

^a^One outlier removed.

^b^Six subjects were not sampled at peak impairment.

^c^Four subjects were not sampled at peak impairment.

### Detection of key cannabinoids in breath

While the results of this study show that measuring ∆^9^-THC by itself in blood or exhaled breath cannot be used as a reliable indicator of recent cannabis use within the impairment window, there are other cannabinoids that may serve as key indicators of recent use through inhalation. Exhaled breath samples were collected from a group of 44 subjects before and after smoking cannabis and analyzed for cannabinoid content. Table 5 shows the percent positivity of six cannabinoids prior to smoking (baseline), within the first 60 min post-smoking (peak impairment window), and more than 60 min post-smoking in subjects’ exhaled breath samples. In particular, CBN, CBC, CBG, and ∆^9^-THCV all had a much greater incidence in breath during the peak impairment window compared to pre-smoking. Interestingly, both CBC and ∆^9^-THCV (shown in bold) were detected in breath only during the peak impairment window. The detectable concentration ranges for these cannabinoids at baseline were < LOQ to 4.1 ng/filter (∆^9^-THC), 0.7 ng/filter (CBN; 1 subject), < LOQ (CBG), and < LOQ to 0.5 ng/filter (CBGA). Within the first hour post-smoking, the concentration ranges were 17.4 to 4364 ng/filter (∆^9^-THC), < LOQ to 694 ng/filter (CBN), < LOQ to 674 ng/filter (CBC), 0.2 to 1064 ng/filter (CBG), < LOQ to 7.4 ng/filter (CBGA), and < LOQ to 77 ng/filter (∆^9^-THCV). Beyond the first hour post-smoking, the concentration ranges were < LOQ to 4.06 ng/filter (∆^9^-THC), < LOQ to 0.5 ng/filter (CBN), 0.2 ng/filter (CBG; 1 subject), and < LOQ to 1.6 ng/filter (CBGA).

**Table 5:** Presence of key cannabinoids in exhaled breath before and after smoking.

Cannabinoid parameters	Percent (%) positivity		
	Baseline (Pre-smoking)^a^	≤ 60 min after smoking	> 60 min after smoking^b^
∆^9^-THC^c,d^	23/34 (67.6%)	40/40 (100%)	37/40 (92.5%)
CBN^d^	1/34 (2.9%)	37/40 (92.5%)	4/40 (10.0%)
**CBC**^d^	**0/34 (0%)**	**39/40 (97.5%)**	**0/40 (0%)**
CBG^d^	2/34 (5.9%)	37/40 (92.5%)	1/40 (2.5%)
CBGA^d^	4/34 (11.8%)	18/40 (45.0%)	4/40 (10.0%)
**∆**^**9**^**-THCV**^**d**^	**0/34 (0%)**	**36/40 (90.0%)**	**0/40 (0%)**

^a^Pre-smoking samples were not collected from 10 subjects.

^b^No data beyond 60 min post-smoking in 4 subjects.

^c^Although ∆^9^-THC was not detected in approximately one-third of subjects prior to smoking, other indicators of prior cannabis use, e.g., ∆^9^-THCA, were detected at baseline in all subjects.

^d^For CBN, CBC, CBG, CBGA, and ∆^9^-THCV, percent positivity differences were significant (*P* < 0.01) when comparing baseline to ≤ 60 min post-smoking and ≤ 60 min post-smoking to > 60 min post-smoking. For ∆^9^-THC, the percent positivity differences were significant (*P* < 0.01) when comparing baseline to ≤ 60 min and > 60 min post-smoking.

## Discussion

Previous studies have failed to demonstrate a clear relationship between impairment and specific concentrations of ∆^9^-THC in blood or oral fluid^2–6^. In agreement with these studies, the results of the present work showed that a majority of a group of 30 test subjects had pre-smoking ∆^9^-THC blood concentrations that exceeded the legal limits currently in place in five U.S. states (Illinois, Montana, Ohio, Nevada, and Washington), in the absence of impairment. The results also showed that post-smoking duration of impairment appeared to be inversely related to baseline blood ∆^9^-THC concentrations, and that subjects with the shortest duration of impairment tended to have the lowest incidence of HGN three hours post-smoking. These findings provide further evidence that single measurements of specific ∆^9^-THC blood concentrations do not correlate with impairment, and that the use of per se legal limits for ∆^9^-THC is not scientifically justifiable at the present time.

Although it may seem counterintuitive, the inverse relationship between impairment duration and baseline ∆^9^-THC blood concentration observed in the present study is consistent with what would be expected from chronic cannabis users who have developed a high degree of tolerance to the impairing effects of ∆^9^-THC. Ramaekers et al. showed that neurocognitive performance was significantly impaired after smoking in occasional cannabis users compared to chronic, heavy users, indicating the development of tolerance^13,14^. The development of tolerance may involve multiple pharmacodynamic mechanisms including the downregulation and desensitization of CB_1_ receptors in various brain regions^15–17^ and the recruitment of alternate neural networks to compensate for the impairing effects of ∆^9^-THC during the performance of neurocognitive tasks^18,19^. It has also been shown that the development of tolerance to ∆^9^-THC may reduce the sensitivity of standardized field sobriety tests in the detection of cannabis impairment^20^. It is therefore not surprising that the subjects in present study, most of whom were chronic, daily cannabis users, presented with high blood concentrations of ∆^9^-THC prior to smoking, in the absence of impairment, that the duration of impairment tended to be shorter in subjects with higher baseline ∆^9^-THC concentrations, and that the lowest incidence of HGN at three hours post-smoking tended to be observed in subjects with the shortest duration of impairment, all of which are indicative of the development of tolerance.

When ∆^9^-THC concentrations in exhaled breath were compared to those in blood at baseline and during peak impairment after smoking, increasing blood concentrations were generally associated with increasing breath concentrations. This result in exhaled breath at baseline in the absence of impairment suggests, just as is the case with ∆^9^-THC concentrations in blood, single measurements of ∆^9^-THC in breath cannot be used to establish impairment. Our findings are consistent with others who have shown that ∆^9^-THC can be detected in breath up to several days since last use^9,10^. Because the leading technologies for breath-based testing for recent cannabis use^9,21^ rely solely on the detection of ∆^9^-THC, this could potentially result in false positive test outcomes due to the presence of ∆^9^-THC in breath outside of the impairment window. It may be that other cannabinoids such as ∆^9^-THCV and CBC, which were detected in breath only during the impairment window in the present study, are more suitable key indicators of recent cannabis use associated with impairment.

Limitations of the present study include the use of cannabis flowers with ∆^9^-THC potencies ranging from 8.5 to 28.4% and the lack of confirmation that subjects had not used cannabis within 12 h of participating in the study. While the range of potencies may have led to a lack of dose control and a high degree of variability in consumption, cannabis users tend to smoke to achieve the desired effect, and thus will compensate by using more of a less potent chemovar to achieve the desired level of intoxication. Most of the subjects in this study were chronic daily users, and all of them experienced some impairment to the level where they felt they could no longer safely drive a car, which was the desired effect. Although specific confirmation of whether subjects had abstained from cannabis use for at least 12 h was not performed, baseline (pre-smoking) samples of blood and breath were collected and subjects were evaluated for nystagmus prior to smoking. Analysis results of these samples were consistent with prior cannabis use, but not recent enough to cause impairment, and the incidence of nystagmus prior to smoking was very low, all of which suggest that the subjects were being honest in their self-reports. All subjects were compensated for their participation and had no reason to be dishonest. While the possibility that some subjects violated the required period of abstinence cannot be ruled out, none of the subjects exhibited evidence of impairment, including nystagmus, prior to smoking.

In conclusion, we present further evidence that single measurements of ∆^9^-THC in blood cannot establish impairment, that single measurements of ∆^9^-THC in exhaled breath likewise do not correlate with impairment, and that ∆^9^-THCV and CBC may be key indicators of recent cannabis use through inhalation within the impairment window.

## Materials and methods

### Clinical study

A total of 74 subjects were recruited to perform a study designed to develop a test that confirms recent use of inhaled cannabis within the impairment window as previously described^11^. All subjects received financial compensation for their participation. Subjects were recruited by disclosing the study through word of mouth to known marijuana users, who then volunteered for the study. The study was performed under a clinical protocol approved by the Cancer Immunotherapy Research Institute IRB (assurance #FWA00029851), and all research activities were conducted in accordance with the Declaration of Helsinki. Written informed consent was obtained from all subjects prior to their participation, and a copy of the signed informed consent form was provided to each subject.

#### Inclusion criteria

To be included, a subject must have been a male or female cannabis user at least 21 years of age. Prior to their scheduled participation, they must have used within the previous 24 h, but not within the last 12 h. Upon entry, subjects were asked to complete a questionnaire requesting their age, sex, race, height, weight, cannabis use history (time since last use, number of days used in the last 14 days, how often they use cannabis, number of years of cannabis use), their primary route of cannabis use, whether or not they use tobacco and alcohol, and any medications or supplements they are taking.

#### Cannabis administration

Each subject was given a single cannabis cigarette and instructed to smoke as much of it as possible within a 10-min period. Cigarettes containing 500 mg of dried cannabis flower with a Δ^9^-THC content ranging from 8.5 to 28.4% were prepared immediately before each smoking session. Cannabis supplies were legally obtained from licensed retail establishments in the Sacramento, CA region. A wide variety of chemovars was included to account for the variability in potencies available in numerous cannabis retail establishments in the various U.S. states where recreational and/or medicinal cannabis has been legalized.

#### Blood draw schedule

Blood samples were obtained from all 74 subjects. To establish baseline cannabinoid levels, capillary blood samples were collected prior to smoking. Post-smoking blood samples were collected immediately after smoking and then at 20, 40, 60, 80, 100, 120, 140, 160, 180, and 200 min post-smoking. Capillary blood (50–100 μL) was collected into BD Microtainer tubes containing lithium heparin anticoagulant (Thermo Fisher Scientific; Waltham, MA) after pricking subjects’ fingers using 17-gauge lancets (McKesson Medical-Surgical Inc., Richmond, VA). Some capillary blood samples were drawn using automated collection devices from Tasso, Inc. (Seattle, WA) and Seventh Sense Biosystems, Inc. (Medford, MA) equipped with sample reservoirs containing lithium heparin. These devices are designed to draw approximately 100–150 μL of whole blood over a period of 1–3 min.

#### Breath collection schedule

The first 30 subjects (#1–30) had only blood samples collected because the original study design was to develop a blood-based cannabis recent use test. Data from these subjects showed that an additional component, exhaled breath, was needed to more accurately detect recent cannabis use within the impairment window. Therefore, breath and blood samples were obtained from a total of 44 additional subjects (#31–74).

To establish baseline cannabinoid levels, breath samples were collected prior to smoking. Post-smoking breath samples were collected immediately after smoking, and then at 10, 20, 30, 40, 50, 60, 80, 120, 180, and 240 min post-smoking in the first 35 subjects. In the last nine subjects, back-to-back breath samples were collected at 20 and 40 min post-smoking. Breath sample collection devices were provided by Sensabues AB (Stockholm, Sweden). These self-contained, single-use devices contain an electrostatic polymer filter and are designed to collect about 20 L of exhaled breath through normal breathing. During sample collection, subjects were seated and instructed to blow through the device until the attached bag was fully inflated. The time required for sample collection was approximately 2–3 min. No instances of hyperventilation or other breathing abnormalities were observed. Devices were kept sealed in their original packaging until immediately before use to prevent contamination and used according to the manufacturer’s instructions. The smoking room was well ventilated and allowed to clear for at least 24 h prior to each subject smoking session. Immediately after sample collection, the devices were resealed, removed from the collection area, and held at room temperature (20–25 °C). All samples were extracted and analyzed within 24 h of collection.

#### Self-assessment of impairment and duration of impairment

All 74 subjects were asked to self-assess their level of impairment before smoking and at each designated time point after smoking based on a scale ranging from 0 (no impairment) to 10, which denoted maximal impairment (incapacitation) for that individual. To normalize, impairment data were expressed as a percentage relative to each individual subject’s maximum reported impairment level. Duration of impairment was determined by the time point post-smoking at which each subject last reported any impairment; for example, if a subject last self-reported impairment at 80–120 min post-smoking, their duration of impairment was two hours.

#### Physical assessment of impairment: HGN

In this study, a subset of 44 subjects were evaluated for HGN as a physical indicator of impairment. In this particular test, subjects are asked to keep their head still and follow a slowly moving horizontal object positioned in front of their face using their eyes only. Both eyes are observed for lack of smooth pursuit, nystagmus at maximum eye deviation (45°), and the onset of nystagmus prior to a 45° deviation. The presence or absence of resting nystagmus is also noted.

### Analytical methods

#### Chemicals and reagents

Six of the seven cannabinoid analytes [∆^9^-THC, cannabinol (CBN), cannabigerol (CBG), cannabigerolic acid (CBGA), ∆^9^-tetrahydrocannabinolic acid A (∆^9^-THCA), and ∆^9^-tetrahydrocannabivarin (∆^9^-THCV)] and the internal standard (IS; ∆^9^-THC-D_3_) were obtained as certified reference materials (CRMs) manufactured by Cerilliant (Round Rock, TX). Cannabichromene (CBC) was obtained as a CRM from Cayman Chemical (Ann Arbor, MI). When not in use, concentrated stock solutions of these agents and working solutions made therefrom were stored at –20 °C.

Acetonitrile, formic acid, methanol, and *n*-hexane were purchased from Thermo Fisher Scientific and were of LC/MS grade. Ethyl acetate (Acros Organics) was purchased from Thermo Fisher Scientific and was of spectroscopy grade (> 99.5%). High purity water (18.2 MΩ) required for preparing the mobile phase and for sample extraction was produced using an EMD Millipore Simplicity water purification system. When not in use, these agents were stored at room temperature (20–25 °C). Nitrogen (N_2_), supplied as a cryogenic liquid in a 230L dewar at a purity of 99.998%, or as compressed nitrogen gas at a purity of 99.999% in T-type cylinders, was obtained from Praxair (Danbury, CT).

#### Analysis of cannabinoids in exhaled breath

A previously validated LC-HRMS analytical method for the quantification of the cannabinoids ∆^9^-THC, CBN, CBC, and ∆^9^-THCV in exhaled breath was used for the analysis of study samples. Additional cannabinoids analyzed included ∆^9^-THCA, CBG, and CBGA. For the preparation of calibration standards, sufficient quantities of the matrix (breath collection devices) were obtained from SensAbues AB. Breath collection devices were kept at room temperature (20–25 °C) within their original packaging to prevent contamination.

Concentrated standard calibration solutions were prepared in methanol at 37.5, 75, 150, 375, 750, and 1500 ng/mL of all cannabinoids combined. Following extraction and reconstitution, final standard concentrations were 2.5, 5.0, 10, 25, 50 and 100 ng/mL, equivalent to approximately 0.2, 0.4, 0.8, 1.9, 3.8, and 7.5 ng/breath filter. The IS solution was prepared in methanol at a concentration of 75 ng/mL. To prepare calibration standards for extraction, 5 µL of the IS working solution and 5 µL of the appropriate calibration standard solution were added directly onto the corresponding filter pad inside the breath collection device. After extraction, the final concentration of the IS was 5 ng/mL (75 µL final volume). Study samples were prepared by spiking with 5 µL IS solution.

Extraction of cannabinoids from breath collection devices was performed as previously described^11^. Briefly, a total of 7 mL methanol were aliquoted through each device and filter housing into glass sample collection tubes. The sample breath collection devices were then removed and the glass tubes were placed in an N-Evap Model 112 analytical nitrogen evaporator (Organomation Associates, Berlin, MA). The eluate was evaporated to dryness under a gentle stream of nitrogen gas, with the water bath temperature set to approximately 50 °C. After evaporation, the samples were cooled to room temperature and reconstituted in 75 µL of a solution containing 75% acetonitrile and 25% water with 0.1% formic acid. The samples were then transferred to a glass microinsert-equipped autosampler vial and placed in the autosampler compartment for analysis according to the method. The chromatographic conditions for the analysis of cannabinoids in exhaled breath were the same as previously described^22^. The assay LOQ was 0.2 ng/filter.

#### Analysis of cannabinoids in blood

Extraction and analysis of ∆^9^-THC and other cannabinoids in whole blood was performed according to a validated method as previously described^22^. Briefly, 50 µL of each sample was mixed with 100 µL of high-purity water in a 1.5-mL microcentrifuge tube and spiked with 5.0 µL of IS solution. To extract, 500 µL of a solution containing 90% *n*-hexane and 10% ethyl acetate (v/v) was added to each sample, followed by vortexing for 30 s. Samples were then centrifuged at 9,300 rcf for 10 min. The supernatant was transferred to a 16 mm × 125 mm glass tube and evaporated to dryness under a gentle stream of nitrogen at 50 °C. Samples were then reconstituted in 75 µL of a solution composed of 65% acetonitrile, 35% water, and 0.1% formic acid and analyzed by LC-HRMS. Supplies of whole blood needed to prepare calibration standards were obtained from a reliable, cannabis-free donor and kept refrigerated (2–8 °C) for up to six weeks. The assay LOQ was 1.0 ng/mL.

The LC-HRMS system consisted of a Thermo Scientific Vanquish ultra-high-performance liquid chromatography (UHPLC) system and a Thermo Scientific Q Exactive mass spectrometer. All analytical data were collected and processed using TraceFinder version 4.1 software (Thermo Fisher Scientific). The mass spectrometer and UHPLC system were configured as previously described^22^.

### Statistical methods

Subject age and cannabis use history are expressed as mean ± standard deviation. The difference in the incidence of HGN at three hours post-smoking between subjects with a one-hour duration of impairment and subjects with a duration of impairment > 3 h was evaluated using an independent samples Student’s t-test with a two-tailed distribution and significance level of 0.05. The correlation between duration of impairment and baseline ∆^9^-THC concentrations, and the correlation between blood and breath ∆^9^-THC concentrations at baseline and peak impairment, was assessed by Pearson correlation analysis. Differences in percent positivity of key cannabinoids in breath before and after smoking were analyzed by one-way ANOVA with Bonferroni’s multiple comparisons post-test. All computations were performed using Graph Pad Prism version 5 (La Jolla, CA) and Microsoft Excel version 16 (Redmond, WA) software.

## Data Availability

All datasets generated and/or analyzed during the present study are either available in the main text and supplementary materials, or can be obtained from the corresponding author on reasonable request.
